# Intranasal LPS-Mediated Parkinson’s Model Challenges the Pathogenesis of Nasal Cavity and Environmental Toxins

**DOI:** 10.1371/journal.pone.0078418

**Published:** 2013-11-08

**Authors:** Qing He, Wenbo Yu, Jianjun Wu, Chan Chen, Zhiyin Lou, Qiong Zhang, Jian Zhao, Jian Wang, Baoguo Xiao

**Affiliations:** 1 Institute of Neurology, Huashan Hospital, Institutes of Brain Science and State Key Laboratory of Medical Neurobiology, Fudan University, Shanghai, China; 2 Department of Neurology, Shanghai Jiaotong University Affiliated First People’s Hospital, Shanghai, China; 3 Department of Physical Test, Nanjing Medical University, Affiliated Nanjing Brain Hospital, Nanjing, China; 4 Department of Neurology, Huashan Hospital, Fudan University, Shanghai, China; 5 Department of Neurology, Xinhua Hospital Affiliated To Shanghai Jiaotong University School of Medicine, Shanghai, China; Virginia Commonwealth University, United States of America

## Abstract

Accumulating evidence implicates the relationship between neuroinflammation and pathogenesis in idiopathic Parkinson's disease (iPD). The nose has recently been considered a gate way to the brain which facilitates entry of environmental neurotoxin into the brain. Our study aims to build a PD model by a natural exposure route. In this report, we establish a new endotoxin-based PD model in mice by unilateral intranasal (i.n.) instillation of the lipopolysaccharides (LPS) every other day for 5 months. These mice display a progressive hypokinesia, selective loss of dopaminergic neurons, and reduction in striatal dopamine (DA) content, as well as α-synuclein aggregation in the SN, without systemic inflammatory and immune responses. This new PD model provides a tool for studying the inflammation-mediated chronic pathogenesis and searching for therapeutic intervention in glia-neuron pathway that will slow or halt neurodegeneration in PD.

## Introduction

Parkinson's disease (PD) is a progressive neurodegenerative disorder affecting 3% of the population over the age of 65 [1]. The cause of gradual loss of dopaminergic neurons and progression of the chronic disease is still unknown. Therefore, an ideal animal model mimicking clinical and pathological features of PD is essential to investigate the pathogenesis and intervention of PD. At present, the available animal models of PD include neurotoxin-based PD models produced by exposure to toxins such as 1-methyl 4-phenyl 1,2,3,6-tetrahydropyridine (MPTP), 6-hydroxydopamine (6-OHDA), rotenone, paraquat and transgenic models [2]. However, all of them have their own advantages and limitations. Some of the cellular and behavioral deficits seen in the humans could be found in 6-OHDA model, but it fails to develop the pathological Lewy bodies (LBs) or α-synuclein-positive inclusions. 6-OHDA can’t pass the blood-brain barrier, the local injection in the substantia nigra (SN) or medial forebrain bundle or striatum is needed to damage dopaminergic neurons, thereby requiring a stereotaxic instrument and intense technical training. Although MPTP can be systemically administered, but it does not exist under natural conditions, the resulting pathogenesis is not comparable to the human disease process, particularly in terms of a highly toxic risk of MPTP to researchers and environment [3]. Importantly, neurotoxin-based PD models deplete dopaminergic neurons directly, making it difficult to study the pathological process and intervention sites. In addition, these models are acute or sub-acute, and can’t represent the chronic and progressive degeneration in PD. Transgenic models focus on some single gene such as α-synuclein, parkin, LRKK2 to study specific molecular events, while the vast majority PD cases are idiopathic, affected by multifactorial factors [4], which can’t be replicated in these models.

Numerous studies have revealed an essential role for microglia activation and neuroinflammation in PD pathogenesis [5]. The activation of microglia in PD patients is observed using positron emission tomography and [(11) C] (R)-PK11195 [6]. Microglia activation and inflammatory factors in brain microenvironment are associated with degeneration of neurons in the SN of PD patients and various PD models [5]. Lipopolysaccharide (LPS), as a potent glial activator, has been used for the induction of dopamine (DA) neurodegeneration through intracranial and intraperitoneal injection [7]. However, these PD models have not been widely adopted and used, primarily because single intracranial injection of LPS is insufficient to induce degeneration and loss of dopaminergic neurons in the SN and systemic LPS challenge did not provide a stable and uniform PD model, or even failure [8].

Recent studies show that humans are often exposed to LPS suspending in the air as a component of the air pollutant PM2.5 or as part of house dust and aerosols generated from contaminated water [9,10]. PM2.5 are fine particles of less than 2.5 μm from suspended particulate matter in air pollutants, commonly originate from oil refineries, metal processing facilities, tailpipe and brake emissions, residential fuel combustion, power plants, and wild fires and composed of both organic and inorganic compounds, including sulfates, nitrates, carbon, ammonium, hydrogen ions, lipopolysaccharide (LPS), metals, and water. Furthermore, occupational exposure to LPS is common for people in agricultural settings or in textile mills [11]. The intranasal (i.n.) LPS exposure is a direct route of communication between the environment and the brain. Airborne infectious, allergic and pollution agents could enter the brain via the nose and bypass the blood brain barrier [12]. Intriguingly, it has been recently postulated that PD might be a primary disorder of olfaction, in which smell loss precedes motor symptoms by years. Staging of brain pathology related to sporadic PD indicate that anterior olfactory nucleus is initially lesioned in PD progression [13]. Therefore, the presence of smell loss and olfactory bulb pathology, together with evidence that airborne neurotoxins or infectious viewed as disease risk factors can enter the brain via the olfactory mucosa, has led to the olfactory vector hypothesis that PD disorders may be caused or catalyzed by agents that enter the brain via the nose [14].

In this study, we created a chronic, progressive mouse model of PD by intranasal instillation of a relatively low dose of LPS (10 µg) every other day for 5 months in 8-month-old C57/B6 mice. These mice display a progressive hypokinesia, selective loss of dopaminergic neurons, and reduction in striatal DA content, as well as α-synuclein accumulation and aggregation in the SN, providing a microglia-neuron pathogenetic process of PD and a time window for the application of potential neuroprotective therapies. 

## Materials and Methods

### Animal and treatment

Female C57BL/6 mice, 8 months old and 20–22g, were purchased from Shanghai SLAC Laboratory Animal Company (Shanghai, China). All experiments were conducted in accordance with the guidelines of the International Council for Laboratory Animal Science. The study was approved by the Ethics Committee of Huashan Hospital of Fudan University, Shanghai, China. All mice were housed under pathogen-free conditions, received food and water ad libitum, and maintained in a reversed 12:12-h (h) light/dark cycle in a temperature-controlled room (25±2°C) for 1 week prior to experimental manipulation. LPS (1mg⁄ml, Sigma-Aldrich, USA) was dissolved in saline solution just before use. Intranasal instillation was performed as described elsewhere [15]. In order to better observe the loss of dopaminergic neurons in the SN in individual mice, we adopted a unilateral intranasal administration of LPS, thereby establishing two controls, that is saline control and contralateral non-LPS control. After a slight anesthesia with ether, mice were held by the neck and were laid upside down with a finger under the neck to limit liquid flow down the trachea. 10 µl of LPS or saline solution was slowly introduced with a micropipette (over~15s) into one nasal cavity. Afterwards, the mice were immobilized in this position for ~10s by slightly to the right side to prevent LPS infiltration to the left nasal cavity. This procedure was repeated every other day for 5 months. 

### Open-field test

Motor behaviour was analyzed in an open-field test. The apparatus consisted of a square (30*30 cm) with a surrounding wall (height 15 cm). The square floor was divided in 16 small squares (7.5*7.5 cm) using transverse and longitudinal segments. Mice were placed in the centre of the structure and their spontaneous activity was analyzed for a period of 30 min, the first 5 min serving as a period of habituation to reduce novelty-induced stress. The apparatus was washed out after each test. We scored the ambulatory episodes, counts and distance traveled.

### Adhesive removal test (ART)

Adhesive removal test (ART) was adapted in rat and mouse models of PD [16]. Prior to test, two training trails were performed by placing adhesive dots on the plantar surface of both forelimbs with equal pressure, which decreases their anxiolytic responses during formal experiment. At the 5th month after LPS or saline instillation, adhesive dots (0.6 cm diameter) were placed on both forelimbs and the time to remove the dot from each forelimb was recorded, with a maximum of 120 s. The order of placement of the adhesive dots (right or left) was alternated between each animal and each session. Median data were calculated across three trails.

### Morris water maze (MWM)

Spatial learning and memory were assessed using the standard MWM, consisting of a circular pool (136 cm diameter×60 cm height) filled with opaque water (pulverized chalk added to the water). The water temperature was maintained at 20±1°C and the room temperature was kept at 21±1°C. The maze was virtually divided into four arbitrary, equally spaced quadrants delineated by the cardinal points: north (N), east (E), south (S) and west (W). A squared Plexiglas platform (13×13 cm) was hidden 0.5 cm below the surface of water in the middle of the NE quadrant (target quadrant). The swim paths were monitored by means of a video computer-based storing system that allowed the automatic calculation of the swim distance (cm), latency (s) and swim speed (cm/s).

The acquisition procedure of the spatial learning task was performed for five consecutive days. Each session consisted of placing the mouse in the pool, facing the wall of the tank, at E, S, SW and NE points (8 consecutive trials per day, starting point alternated between each trial. The mouse was allowed 60 s to search for and climb onto the submerged platform. If the animal failed to locate the platform within this delay, it was subsequently placed on the platform by the experimenter. The latencies to platform were measured using the video tracking system (Coulbourn, USA)

### Tissue fixation and freeze-sectioning

Mice were perfused through the left ventricle with saline solution, followed by 4% paraformaldehyde in 0.1 M phosphate buffer, pH 7.4, for 15 min. The brains were removed and cryoprotected by soaking in 10%, 20% and 30% sucrose solution for each day in phosphate buffer until they sank. The brains were obtained and frozen in liquid nitrogen. Parallel series of 10µm-thick coronal sections were obtained in a freezing microtome.

### Immunofluorescence

For immunostaining analysis, non-specific binding was blocked with 3% bovine serum (Serotec, UK), and permeabilized with 0.1% Triton X-100 in 1% BSA-PBS for 30 min. The sections were incubated at 4°C overnight with monoclonal rabbit anti-TH (1:500; Chemicon, USA), monoclonal mouse anti-NeuN (1:500; Chemicon, USA), anti-α-synuclein (1:500; Epitomics, USA), anti-F4/80 (1:1000; Epitomics, USA), anti-CD11b (1:500; eBioscience, USA), p-NF-кB/p65 (1:1000; Chemicon, USA), and then incubated with corresponding secondary antibodies at room temperature for 2 h. The nucleus was stained by Hoechst 33342 (1μg/ml, Sigma-Aldrich, USA). Control sections were run following identical protocols, but omitting the primary antibodies. For each animal, ten sections of brain were examined in a blinded fashion.

### Proteinase K (PK) treatment

For assessing accumulation of insoluble α-synuclein, a PK digestion step was included prior to immunostaining. Briefly, sections were mounted and dried on Permafrost+ glass slides for at least 8hrs at 55°C. Sections were then briefly hydrated with TBS-T (10 mM Tris–HCl, pH 7.8; 100 mM NaCl; 0.05% Tween20), and digested with 50 μg/ml PK (Invitrogen, Carlsbad, CA) in TBS-T (10 mM Tris–HCl, pH 7.8; 100 mM NaCl; 0.1% Tween20) for a period of 1.5 h at 55°C. Sections were then fixed for 10 min using 4% paraformaldehyde and then processed for α-synuclein immunofluorescence as described above.

### Western blot analysis

After removing the brain rapidly, the tissues were separated from the brain and then homogenized on ice with a microcontent motor-operated tissue homogenizer (KIMBLEKONTES, USA) in ice-cold lysis buffer (1×PBS, 1% Nonidet P-40, 0.5% sodium deoxycholate, and 0.1% SDS, RIPA) supplemented with protease inhibitors. Lysates were centrifuged at 10,000 × g for 20 min at 4°C, the supernatants were collected. Protein concentrations were determined by a Bradford protein assay. Equal amounts of protein (30 µg) were separated by SDS-PAGE and electroblotted onto nitrocellulose filter (NC) membrane (Millipore). After non-specific antibody binding was blocked with 5% non-fat dry milk, membranes were incubated at 4°C overnight with polyclonal rabbit anti-TH (1:2000, Millipore, USA), polyclonal rabbit anti-α-synuclein (1:1000, Cayman Chemicals Company, USA), polyclonal rabbit anti-ChAT (1:2000, Chemicon, USA) and monoclonal rabbit anti-GAPDH (1:8000, Epitomics, USA). After washing in TBST, the immunoblots were incubated with horseradish peroxidase-conjugated secondary antibodies (Cell Signaling Technology) for 1 h. The immunoblots were developed with an enhanced chemiluminescence (ECL) reagents (Millipore, USA), and measured with Quantity Software (Bio-Rad, CA). To compare protein loading, antibody directed against GAPDH was used.

### The measurement of striatal biogenic amines

Brains were used to dissect the two striata. The striatum samples were weighed and homogenized in 10 volumes of 0.2 M perchloric acid containing 100 µM EDTA-2Na and 100 ng/ml isoproterenol (the internal standard for the measurement of catecholamine and 5-HT contents) in a homogenizer at a maximum setting for 20 s on ice. After 30 min in the mixture of ice and water, the homogenate was centrifuged at 15 000 × g for 20 min at 4°C. The solution of 1 M sodium acetate was added to adjust the pH value to approximately 3.0. After filtration (0.45 µm), the samples were injected into a HPLC system.

DA, dihydroxyphenylacetic acid (DOPAC), homovanillic acid (HVA), noradrenaline (NE), isoprenaline (Iso), 5-hydroxytryptamine (5-HT) and 5-hydroxyindoleacetic acid (5-HIAA) were detected using a HPLC-electrochemical detection system. Briefly, 20 µL of the homogenate sample was injected into the HPLC-electrochemical detection system by using an L-2200 autosampler (HITACHI, Tokyo, Japan) at 4°C, separated at 25°C on a reverse-phase analytical column (EICOMPAK SC-5ODS, 3.0 mm×150 mm; Eicom, Kyoto, Japan), eluted at a flow rate of 0.5 ml/min at 30°C with 0.1 M sodium acetate/citric acid (pH 5.4) containing 17% methanol, 190 mg/L sodium l-octanesulfonate, and 5 mg/L EDTA-2Na, and determined by an 2465 electrochemical detector (Waters, Milford, MA, USA). The chromatograms were recorded and analyzed using a computer with Millennium32 Chromatography Manager Software (Waters) [17,18].

### Cell viability

To understand whether LPS-induced accumulation and aggregation of α-synuclein in SN can cause antigen-specific immune response, splenic mononuclear cells (6×105/200 ml/well) were incubated for 48 h at 37°C in the presence or absence of α-synuclein (1 μg/ml). Cell viability was detected by MTT assay. Briefly, 100 μl of MTT solution (0.5 mg/ml Duchefa) was added to cultured cells and incubated for an additional 4 h at 37°C, until the media turned purple. Absorbance at 570 nm was measured by a microplate reader after addition of 100 μl DMSO. Each experiment was done in triplicate. Results were expressed as OD value at 570 nm. 

### Nitrite assay

Culture supernatants obtained as above were collected to measured nitric oxide (NO) by Griess reaction. Supernatants (100 μl) of cultured cells were mixed with 100 μl of Griess reagent for 10 min at room temperature. Absorbance was measured at 510 nm in an automated plate reader. Concentrations of nitrite were determined by reference to a standard curve of sodium nitrite (Sigma). Determinations were performed in duplicate. The results were expressed as μM.

### Cytokine ELISA assay

Simultaneously, culture supernatants obtained as above were collected to cytokine concentrations of IL-1β, IL-6, TNF-α, IFN-γ, IL-10 (Pepro tech Inc, USA) and IL-17 (eBioscience, USA) by a sandwich ELISA kits following the manufacturer’s instructions. Determinations were performed in duplicate. The results were expressed as pg/ml.

### Statistical analysis

Comparisons between two experimental groups and both sides were performed using Student’s t test by one-way ANOVA using GraphPad Prism 4 (GraphPad Software, Inc.). Data were presented as mean ± S.E.M. P value less than 0.05 was considered significant. 

## Results

### Behavioral alterations in mice with chronic i.n. LPS instillation

**Figure 1:**
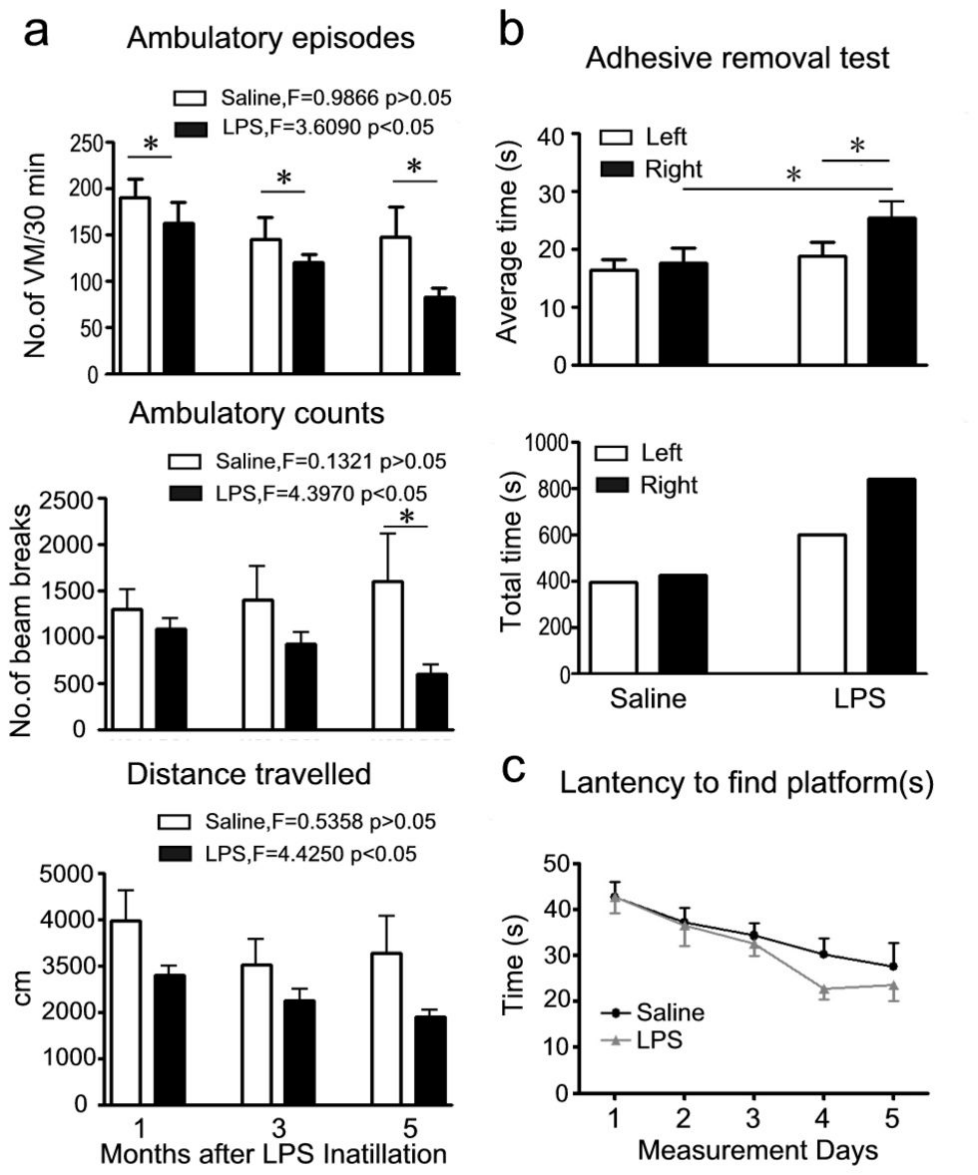
Behavioral alteration in mice with chronic i.n. LPS administration. a) The ambulatory motility (VM=voluntary movement), Motor behavior was analyzed in an open-field test at the 1st, 3rd and 5th months after LPS instillation. b) Adhesive removal test, the asymmetry of left and right movement was detected by the adhesive removal test at the 5th month after LPS instillation. c) Latency to find platforms (from day 1 to day 5), the cognition was measured by Morris water maze at the 5th month after LPS instillation. Left=time to remove adhesive dots from left forelimb; Right= time to remove adhesive dots from right forelimb. Bars indicate SD for 7-10 mice at each point. *P<0.05 compared with the contralateral side and/or saline control, respectively.

To examine the motor activity after LPS instillation in the mice, open-field test was performed at the 1st, 3rd and 5th month. As shown in Figure 1a, LPS-administered mice developed a progressive reduction in ambulatory motor activity with the increase in the number of LPS instillation (Ambulatory episodes, F=3.609; Ambulatory counts, F=4.397; Distance traveled, F=4.425; *P*<0.05, respectively), and a progressive hypokinesia despite not all results reached statistical significance compared with saline-administered mice (Ambulatory episodes, *P*<0.05, respectively at the 1st, 3rd and 5th months after LPS instillation; Ambulatory counts, *P*<0.05, at the 5th month after LPS instillation). Our model exhibits a basic feature of human PD, chronic progressive bradykinesia. 

ART was adapted in rat and mouse models of PD [16]. In our study, ART was performed at the 5th month after LPS instillation to assess left/right symmetrical movement. In saline control mice, the time to remove the adhesive dots from the left and right sides was identical, while in mice challenged with LPS, a significant extension of time to remove adhesive dots from the right side was observed compared with the left side and saline control mice (Figure 1b, *P*<0.05). These data indicated an obvious motor asymmetry in the mice with chronic i.n. LPS instillation. A unilateral model is advantageous as the animals present an internal control in the other brain hemisphere.

Morris water maze (MWM) was used to investigate whether i.n. LPS instillation could affect memory in these mice. The result did not reveal a significant difference between LPS and saline groups. Moreover, the latency to find the platform along the 5-day acquisition procedure gradually decreased in both groups, showing that they learned the task with similar performances (Figure 1c). 

### Neuronal changes in mice with chronic i.n. LPS instillation

**Figure 2:**
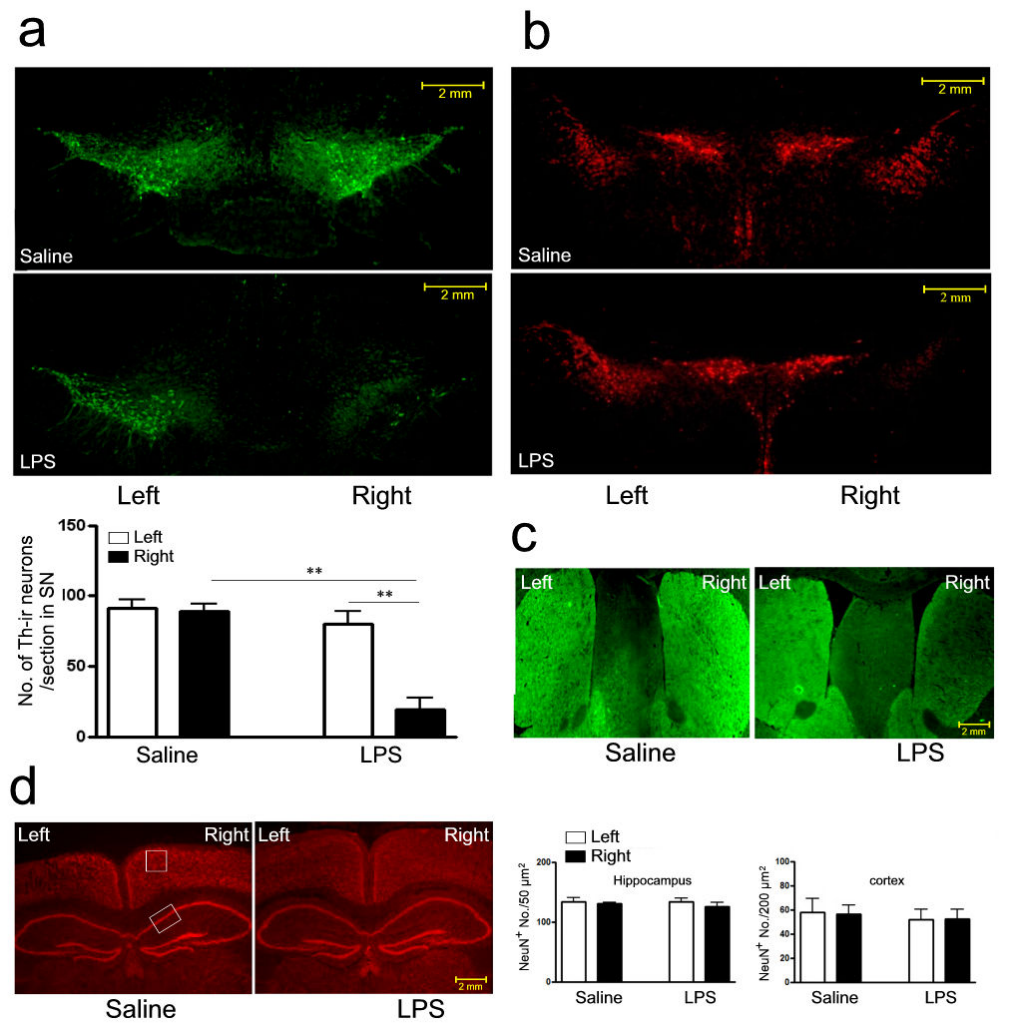
Pathological changes in mice subjected to chronic i.n. LPS administration. a) Loss of dopaminergic neurons in the SN of mice challenged with right intranasal LPS administration of 10 µg/ every other day for 5 months; b) DA neurons in the VTA region on both sides; c) Representative photomicrographs illustrating TH immunoreactivity in the striatum; d) Neurons in the hippocampus and cortex were immunostained with anti-NeuN antibodies. Bars indicate SD for 7-10 mice. **P<0.01 compared with contralateral side and saline control, respectively; scale bar is 2 mm.

The characteristic pathological event of PD model is the loss of dopaminergic neurons in the SN. Next, we also observed the pathological changes in these mice with chronic i.n. LPS exposure. The data showed the mice developed severe loss of tyrosine hydroxylase-immunoreactive (TH-ir) neurons in the right SN of mice 5 months after LPS instillation compared with saline control and contralateral brain. TH-ir neurons were stereologically counted from anterior to posterior of the SN, and found a 79% and 76% reduction in the right SN compared with saline control and contralateral brain (Figure 2a, *P*<0.01, respectively). However, dopaminergic neurons in the adjacent ventral tegmental area (VTA) region were spared of the LPS insult (Figure 2b). The intensity of TH-ir staining was reduced in LPS-treated right striatum than in saline control and contralateral brain (Figure 2c), while NeuN-positive (NeuN^+^) neurons in the hippocampus and cortex were not affected in saline and LPS groups (Figure 2d). 

**Figure 3:**
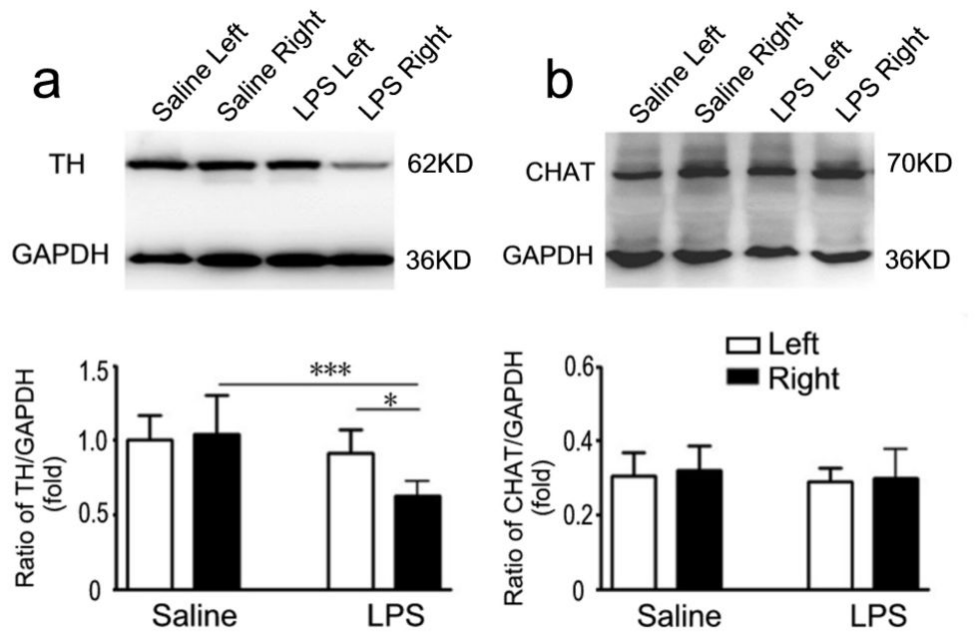
Western blot analyses of TH and ChAT in protein extracts from the left and right sides of the SN in mice challenged with right intranasal LPS (10 µg/every other day) or saline administration of for 5 months. Semi-quantification of relative TH and ChAT protein was obtained from among at least six different animals. *P<0.05 and ***P <0.01 compared with the contralateral SN and saline control, respectively.

Nigral TH protein level was further determined by western blot analysis in the mice after LPS instillation. The result showed about 40% reduction of nigral TH protein compared with saline control and contralateral brain (Figure 3a, *P*<0.001 and *P*<0.05, respectively). In addition, the expression of choline acetyltransferase (ChAT) was identical at both sides within and between LPS or saline groups (Figure 3b). Therefore, this PD model reproduces the signature lesion of PD, the relatively selective degeneration in SN, but not in hippocampus and cortex. Consistent with previous PD models, intranasal LPS-induced neurodegeneration is primarily observed in dopaminergic neurons. Our data implicated a parallel changes between behavioral and pathological alterations. The decreased and asymmetrical motor activity was in concert with the loss of dopaminergic neurons in the SN in the mice with LPS instillation, while the memory function was not affected in these mice in concert with the unaffected cholinergic neurons in the hippocampus and cortex. 

**Figure 4:**
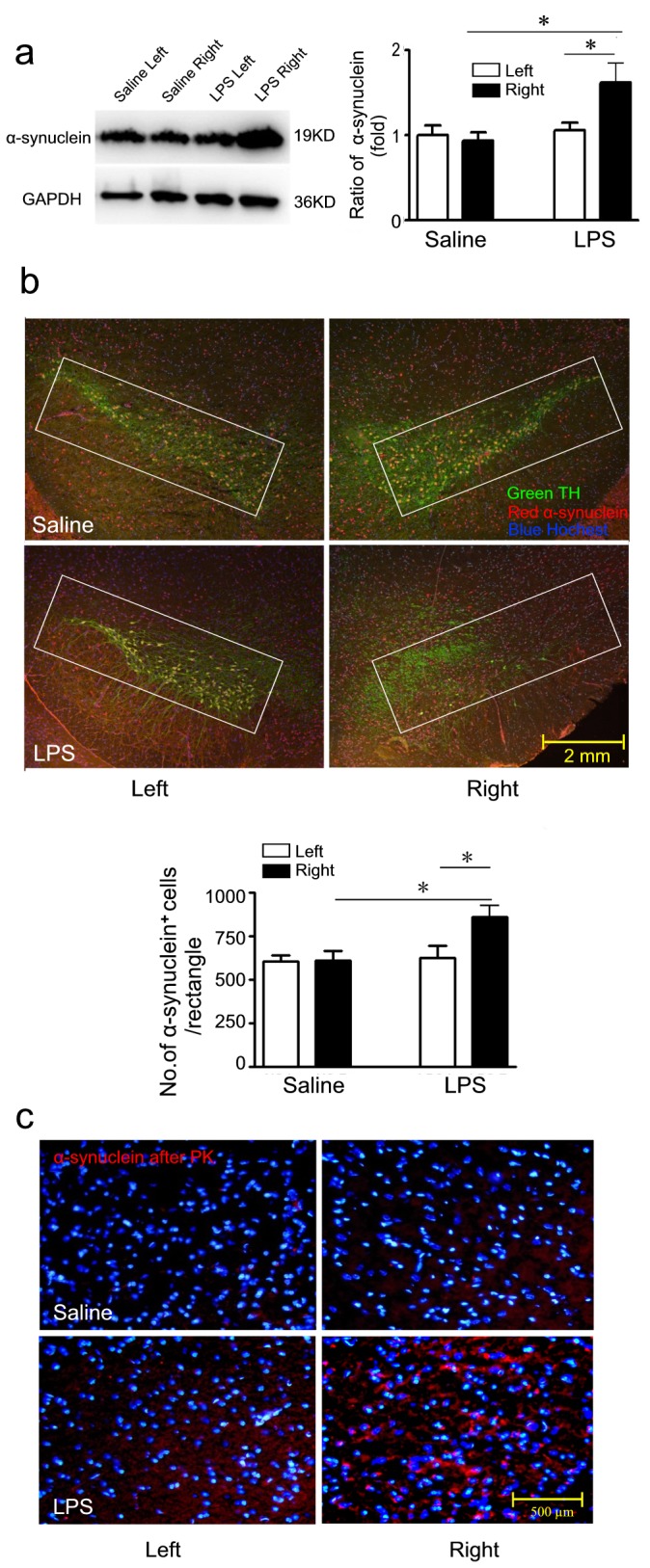
Pathological changes in the olfactory bulb in mice subjected to chronic i.n. **LPS administration**. A) dopaminergic neurons (green) and α-synuclein accumulation (red) around the olfactory bulb in the mice challenged with right intranasal LPS administration of 10 µg/ every other day for 5 months. a) magnification of 4×, scale bar is 2mm; b),c),d) magnification of 10×, d) merge of a,b,c, scale bar is 500 μm; B) Immunohistochemical evidence for CD11b (red) expression in the olfactory bulb of mice challenged with right intranasal LPS administration of 10 µg/ every other day for 5 months. a) CD11b; b) nucleus; c) merged; scale bar is 500 μm.

The olfactory bulb which contains a large population of dopaminergic neurons may be involved in the pathogenesis of PD, thus the thickness of olfactory bulbs layers and its TH-ir neurons were analyzed. As shown in Figure 4A a, b, the loss of TH-ir neurons in the olfactory bulb of mice treated with LPS were obviously reduced, as compared with that of mice treated with saline.

### Accumulation and aggregation of α-synuclein in mice with i.n. LPS instillation

**Figure 5:**
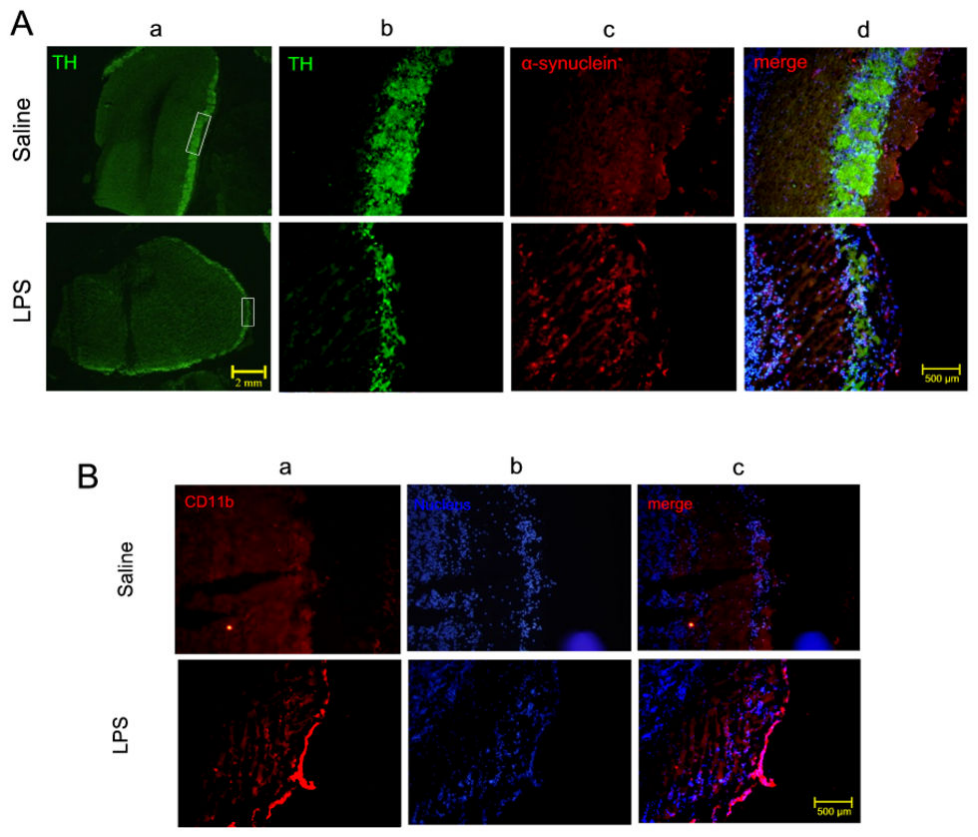
Accumulation and aggregation of α-synuclein were determined by Western blot and immunostaining. a) Western blot, semi-quantification of α-synuclein protein was shown in histogram. b) Three-labeled immunofluorescence (Green=TH; Red=α-synuclein and Blue=Hochest) and quantitative analysis of α-synuclein^+^ cells within the rectangles; scale bar is 2 mm. c) Accumulation and aggregation of α-synuclein in the SN. The expression of insoluble α-synuclein (red) after PK treatment around the nucleus (blue); scale bar is 200 μm. Semi-quantification of α-synuclein protein was obtained from among at least six different animals. *P<0.05 compared with the contralateral SN and saline control, respectively.

Deposition and aggregation of α-synuclein in the LBs is believed to be a hallmark lesion in PD pathology [19]. We next defined whether α-synuclein accumulation was involved in our model by immunofluence and western blots. As shown in Figure 5a and b, the expression of α-synuclein in LPS-challenged right SN was enhanced compared with contralateral brain and saline control at the 5th month after LPS instillation (*P*<0.05, respectively). Figure 5b shows three-labeled immunofluorescence, including TH-ir neurons (green), α-synuclein (red) and nuclear (blue). Quantitative analysis of α-synuclein-positive dots within the rectangles (Figure 5b) was performed with Image-pro Plus software. The number of α-synuclein-positive dots in LPS-challenged right SN was significantly increased compared with contralateral brain and saline control (Figure 5b, *P*<0.05, respectively).

Considering insoluble aggregation of α-synuclein is pathogenic, we examined PK resistant α-synuclein immunostaining on both sides of mice challenged with LPS and saline. PK resistant Lewy-body-like α-synuclein aggregates was obviously observed in the perinuclear compartment of dopaminergic neurons in LPS-treated right SN compared with contralateral brain and/or saline control (Figure 5c), indicating that insoluble α-synuclein may participate in the pathogenesis of DA degeneration in LPS-treated mice. α-synuclein pathology is present in olfactory bulb of PD, and the expression of α-synuclein is observed. As shown in Figure 4A c,d, the expression of α-synuclein in the olfactory bulb of mice treated with LPS obviously increased, as compared with that of mice treated with saline.

### Striatal biogenic amines in mice with chronic i.n. LPS instillation

**Figure 6:**
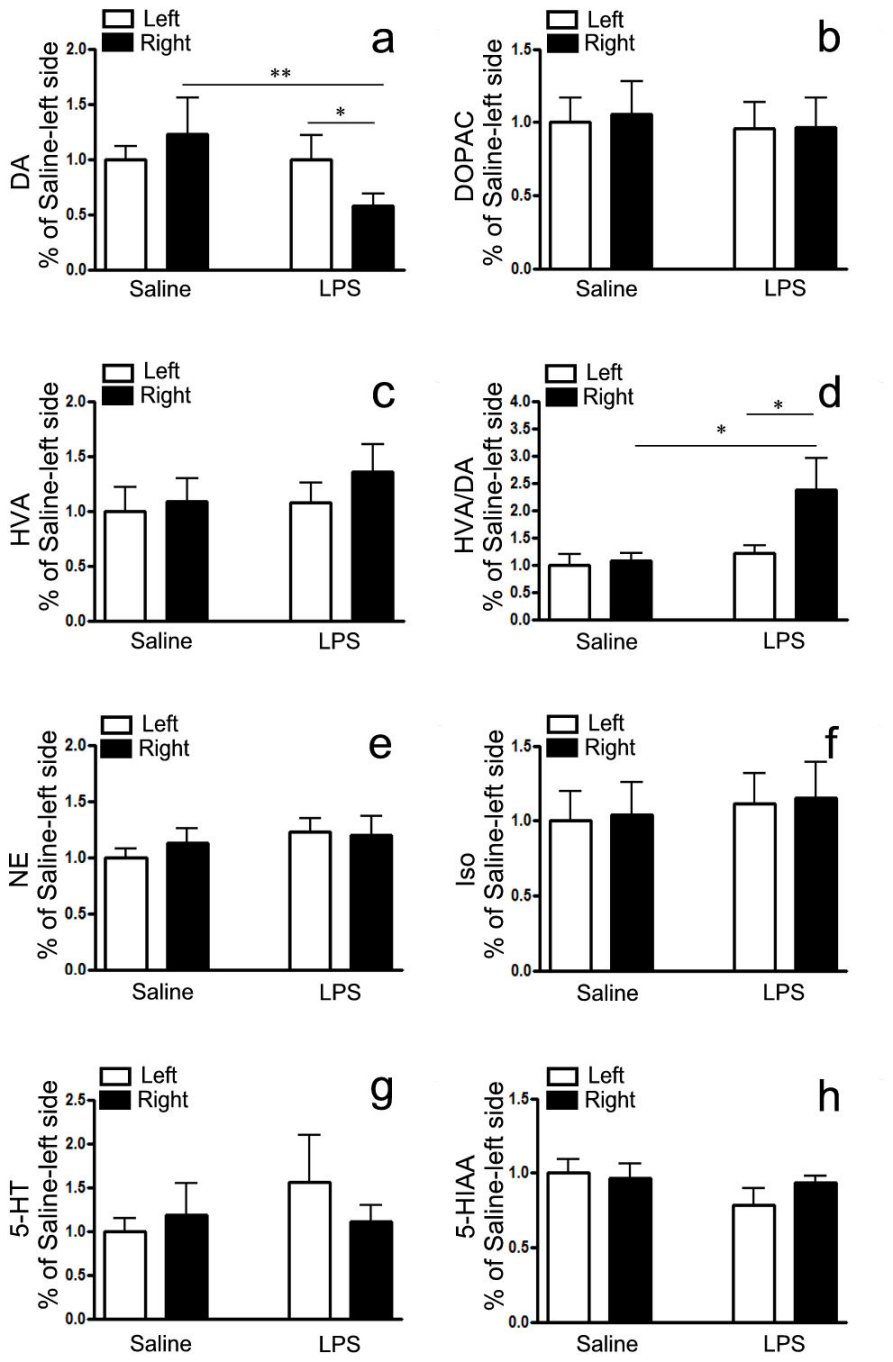
The changes of the striatal biogenic amines following chronic i.n. **LPS in mice**. The levels of DA and its major metabolites DOPAC and HVA, as well as NE, Iso, 5-HT and 5-HIAA in SN were determined by specific HPLC assay at the 5th month after LPS inoculation. a) DA; b) DOPAC; c) HVA and d) the ration of HVA/DA; e) NE; f) Iso; g) 5-HT; h) 5-HIAA. Quantitative results were obtained from among at least six mice. *P<0.05 and **P<0.01 compared with the contralateral striatum and saline control, respectively.

To explore whether i.n. exposure to LPS have effects on the neurotransmitters, the levels of DA and its major metabolites DOPAC and HVA, as well as NE, Iso, 5-HT, 5-HIAA were determined by HPLC assay at the 5th month after LPS instillation. The concentration of DA detected in LPS-treated striatum was significantly lower than in saline control and contralateral brain (Figure 6a, *P*<0.01 and *P*<0.05, respectively), but the levels of DOPAC (Figure 6b) and HVA (Figure 6c) were not altered in LPS-challenged mice. However, dopamine turnover (HVA/DA) in LPS-treated striatum was also significantly higher compared with saline control and contralateral brain (Figure 6d, *P*<0.05, respectively), suggesting that the rate of DA metabolism is rapid in LPS-induced mice. No significant difference was found in levels of NE, Iso, 5-HT and 5-HIAA between and within two groups (Figure 6e, f, g and h). 

### Microglia activation in mice with chronic i.n. LPS instillation

**Figure 7:**
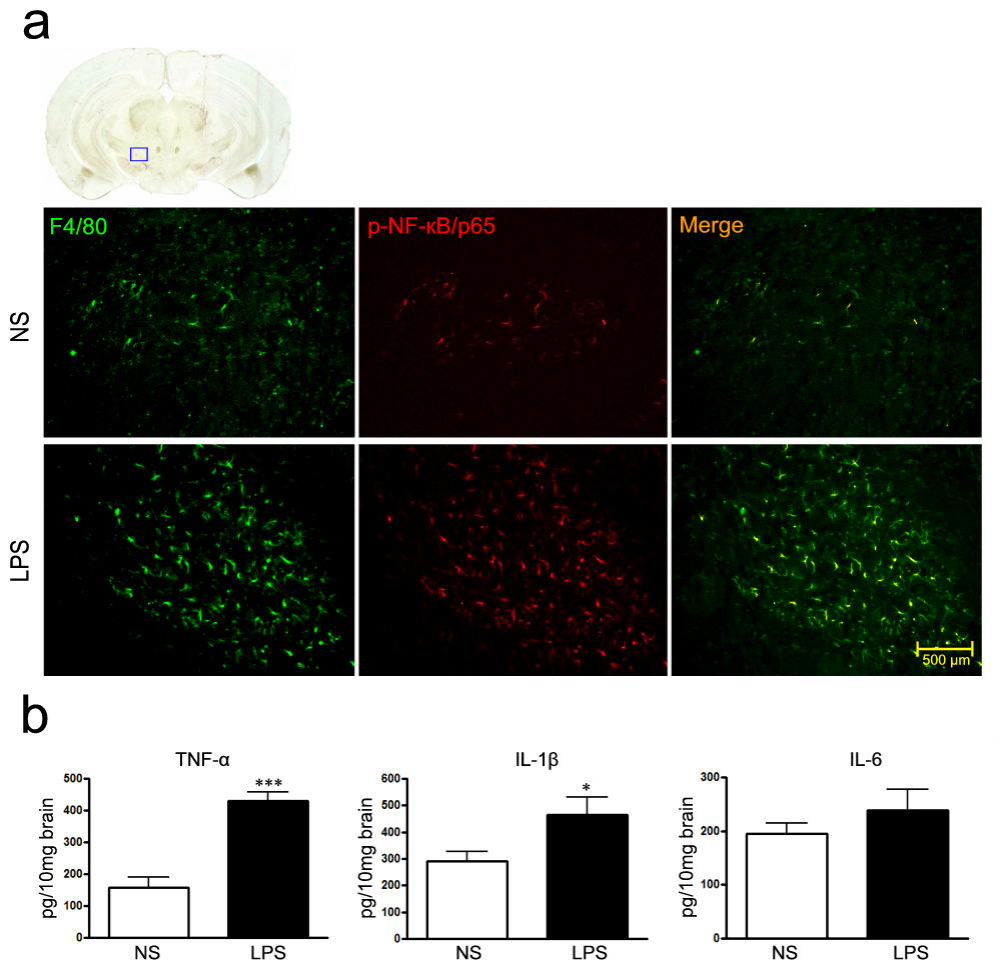
Microglia activation in the SN in mice after chronic i.n. LPS exposure. a) Immunohistochemical evidence for F4/80 (green) and p-NF-**к**B/p65 (red) expression in the SN of mice challenged with right intranasal LPS administration of 10 µg/ every other day for 5 months; Scale bar is 500 µm; b) TNF-α, IL-1β and IL-6 expression in the SN determined by ELISA assays. ***P<0.001, compared with the contralateral side and saline control, respectively; *P<0.05, compared with the contralateral side and saline control.

Since LPS is a potent glial activator, we observed the inflammatory response in the SN after i.n. LPS administration. F4/80 is a mononuclear phagocyte marker, which is solely expressed on the surface of macrophages and serves as a marker for mature macrophage tissues, including Kupffer cells in liver, splenic red pulp macrophages, brain microglia, gut lamina propria, and Langerhans cells in the skin [20,21]. We found F4/80 and p-NF-кB/p65 was greatly up-regulated in LPS-treated right SN compared with contralateral brain and/or saline control (Figure 7a). TNF-α, IL-1β was also released from the same area in these mice (Figure 7b, *P*<0.05, respectively) while IL-6 expression was not changed. 

Because olfactory bulb microglia may respond to LPS challenge, the expression of CD11b, a microglia marker, was detected in the olfactory bulb. As shown in Figure 4B, the expression of CD11b was greatly up-regulated in LPS-treated right olfactory bulb compared with saline control. These results indicated that microglia was activated in both olfactory bulb and SN of mice treated with LPS.

### The influence of peripheral immune in mice with chronic i.n. LPS instillation

**Figure 8:**
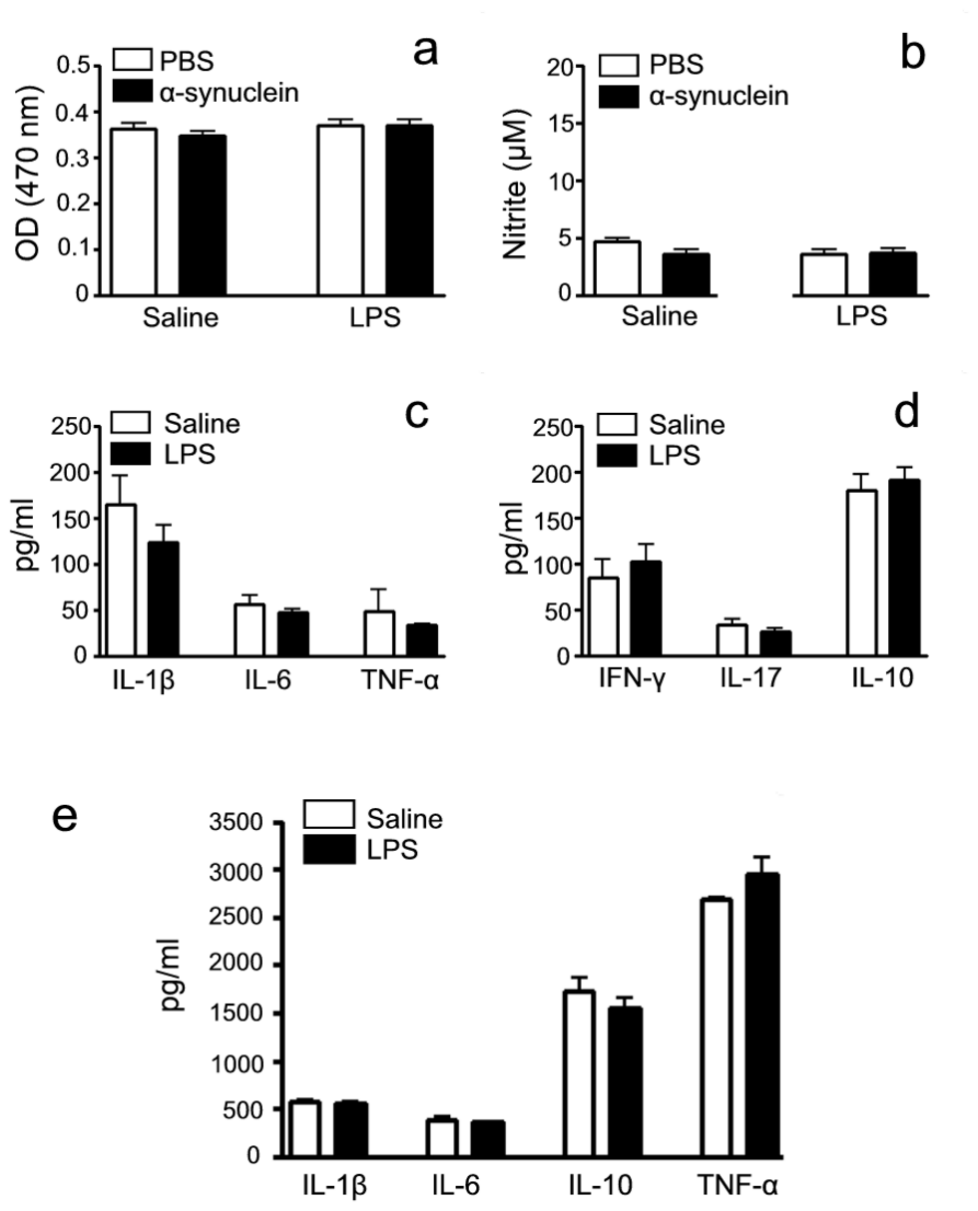
The influence of peripheral immune on mice with chronic i.n. LPS. At the 5th month after LPS inoculation, mice were sacrificed and splenic mononuclear cells and the serum were isolated. The viability of T cells, oxidation products and inflammatory cytokines from spleens were detected in the presence and/or absence of α-synuclein stimulation by MTT, Nitrite and ELISA assays, respectively. Inflammatory cytokines from the serum were detected by ELISA assays. a) The viability of T cells; b) NO production, c) The secretion of inflammatory cytokines IL-1β, IL-6 and TNF-α; d) the secretion of Th1 IFN-γ, Th2 IL-10 and Th17 IL-17 and e) The secretion of inflammatory cytokines IL-1β, IL-6, IL-10 and TNF-α from the serum of the mice. There was no statistical significance between and within two groups. Determinations were performed in duplicate and results were expressed as mean±S.E.M. from at least six mice.

To further explore whether i.n. LPS instillation could cause systemic inflammatory and immune responses, we prepared splenic mononuclear cells and the serum to examine the viability of T cells, oxidation products and inflammatory cytokines at the 5th month after LPS instillation. As shown in Figure 8, there were no any significant differences in both spleen and serum between LPS and saline groups, revealing that i.n. LPS instillation did not influence systemic inflammatory and immune responses. LPS is a potent stimulator of both peripheral immune cells (macrophages and monocytes) and CNS glia (microglia and astrocytes) and causes their release of various immunoregulatory and proinflammatory cytokines and free radicals. In comparison to our LPS i.n. exposure, intraperitoneal LPS injection also induces liver and kidney injury in mice [22,23]. 

## Discussion

To investigate the pathogenesis and intervention of chronic neurodegenerative PD, animal models have been widely used in the past four decades. These models, based on the systemic or local (intracerebral) administration, toxic or transgenic challenge, replicate most of the phenotypic and pathological features of PD. However, they do not adequately present sets of clinical and pathogenic characteristics in human PD [3]. Both toxic and transgenic PD models have their own advantages and limitations. Although toxic models (such as 6-OHDA or MPTP) are excellent animal models, they do not reflect the pathogenic events occurring in human. Importantly, none of toxic models accurately recapitulates the complex and progressive pathology that characterizes human PD. However, transgenic models offer the chronic nature of the degenerative process as well as the distribution of human pathology, but the absence of consistent neuronal damage in the nigrostriatal pathway remains a major limitation for these models [24]. We try to develop PD model that reflects the progressive nature of the disease and its complexity in terms of the extent of pathology and biochemical change for meeting the requirement of translational medicine.

The activation of microglia and increase of proinflammatory factors are believed to contribute to the progressive DA neurodegeneration. LPS, a potent stimulator of micrgolia, has been used to result in a delayed, progressive and selective degeneration of the nigrostriatal DA neurons in rodents, reminiscent of DA neurodegeneration in PD [7]. In addition, LPS doesn’t seem to have a direct effect on neurons most likely due to the lack of TLR4 [25], making it an excellent tool to study inflammation-mediated DA neurodegeneration [26]. In the past few years, LPS-induced PD models have been performed and explored via different routes, such as SN single injection, SN chronic infusion, systemic LPS injection, utero LPS injection and intrapallidal LPS injection. Specific loss of SNpc (Substantia Nigra Pars Compacta) neurons and reduced striatal DA content could be detected in these LPS models [7]. Notably, these PD models have not been widely adopted and used. Several reasons may contribute to this lack of popularity: a) stereotactic injection is a technical deterrent for many laboratories; b) it takes too long to detect nigrostriatal damage with i.p. injection. Although this progressive cell loss can be an attractive feature, it is not feasible for most studies; c) protein aggregation and extranigral pathology have not been reported; d) despite LPS i.p. injection being a useful tool to establish PD model, it also triggers systemic inflammation and liver or kidney injury [22,23].

In this study, LPS was intranasally administered in mice, displaying some key features of PD like the early stage of the human pathology: a) the mild hemilateral hypokinesia; b) a chronic progressive disease course and relatively selective loss of dopaminergic neurons in the nigrostriatal pathway; c) accumulation and aggregation of α-synuclein in SN; d) a change in DA turnover. α-synuclein is found in all LBs and composes of the major component of LBs, suggesting an important role in PD pathogenesis [19]. Very little α-synuclein involvement was observed in 6-OHDA model, MPTP acute model, and transgenic model such as LRRK2 model [4]. In comparison to these models, our model gains advantage in replicating the abnormal accumulation and aggregation of α-synuclein in the SN. These key features of PD were replicated in LPS-challenged right side, but not in saline-treated mice and contralateral brain, indicating the persistent intranasal inflammatory stimulation in mediating chronic PD progression and a direct cause-effect relationship for inflammation-induced development in PD neurodegeneration.

It has been established that up to 85% of the patients suffering from PD show a loss of olfactory function in addition to the classical motor symptoms [27]. One possibility of the association between PD and olfactory dysfunction is that PD might be caused by a virus or chemical agent that enters into the CNS via the nose, activating the immune response of the brain that may lead to neuronal damage [12]. Anatomically, the olfactory nerve is uniquely vulnerable to exogenous agents’ penetration since olfactory receptor cells that make up olfactory nerve are first-order neurons, projecting axons directly to the brain without an intervening synapse. They project their axons to the areas in the brain including diagonal band (HLDB), the substantia nigra (SN), the dorsal raphe (DR), and the locus ceruleus (LC) [14]. Different authors respectively reported the relationship between rhinorrhea and olfaction in PD disease [28], revealing that nasal inflammation may be associated with the pathogenesis of PD. Intranasal exposure to LPS has been proved to induce airway inflammation characterized by neutrophil and macrophage infiltration and the production of chemokines and cytokines including TNF-α [29]. In the olfactory epithelium with inflammation, a corresponding loss of olfactory neurons was observed [30]. Recently, high incidence of immunopositive α-synucleinopathy in the olfactory mucosa was present in the individuals with clinically as well as neuropathologically confirmed PD patients [31], further confirming the importance of the olfactory entry zone in propagation of α-synucleinopathy in the human. Intranasal LPS first arrived at the olfactory bulb, in which dopaminergic neurons represent a fraction of the cells located in the most external (glomerular) layer, maintaining the olfactory sensation [32]. In support with previous studies demonstrating that inhaled toxins could cross the blood brain barrier, and that the damage in Parkinson’s could begin first in the olfactory bulb and then spread from there to the SN [13,14], we looked at TH-ir neurons and α-synuclein in the olfactory bulb and found loss of TH-ir neurons and enhanced α-synuclein expression around the olfactory bulb in LPS-treated mice. Simultaneously, CD11b-positive microglia in the olfactory bulb of mice treated with LPS was obviously higher than that treated with saline. Therefore, it sounds reasonable for our assumption that LPS-induced microglia activation and α-synuclein aggregation in the olfactory bulb could affect the SN inside the brain.

The mechanism underlying intranasal LPS-induced DA neurodegeneration might be primarily attributed to the neuroinflammation in the nigrostriatal region, since that LPS does not seem to have a direct effect on neurons most likely due to their lack of functional expression of Toll-like receptor 4 (TLR4) [25], and it is capable of activating glia, especially microglia to release a wide array of inflammatory and neurotoxic factors including nitric oxide (NO), reactive oxygen species (ROS), cytokines such as IL-1β, IL-6 and TNF-α, as well as cyclooxygenase-2 (COX-2) [5]. Our study proved microglia activation and release of TNF-α and IL-1β in the SN in the mice after chronic LPS exposure. Why does the inflammatory response derived from activated microglia especially attack dopaminergic neurons in SN? Unlike neurons in the hippocampus or cortex, these neurons display exquisite sensitivity to the inflammatory stimuli and oxidative stress [33] and live in a region of the brain reported to have the highest density of microglia [34]. In VTA, dopaminergic neurons were seldom damaged, consistent with previous finding that neurodegeneration occurred in SN in PD patients and classic MPTP model can’t be obviously observed in the VTA region [35].

The content of DA and DA metabolites in PD patients was extensively studied in the 1970s, the correlation between DA content and its metabolism alongside disease progression is still debated [36], mainly due to disease progression, age and gender and the inconsistency of determination. The reduction of DA content depends on the model type. Acute MPTP treatment produced a marked DA depletion, while chronic MPTP induced a reduction in DA levels [37]. HVA in the CSF of PD patients appear not to mirror DA changes precisely. The decrease of HVA was also quantitatively different from DA loss: while DA decreased by about 75%, HVA decreased by only about 25% [36], suggesting a change in DA turnover occurred in course of the disease. In our model, while DA decreased by about 48%, HVA did not exhibit a meaningful change. However, the ratio of HVA/DA in mice challenged with LPS was increased, suggesting that the rate of DA metabolism is rapid. Moreover, NE, Iso, 5-HT and 5-HIAA levels were not affected. These results further define that our PD model is a relatively selective degeneration of dopaminergic neurons. 

In conclusion, an ideal model of PD not only should display the basic characteristics of PD pathology, but also demonstrates the pathological process of the disease development. Our model, being purely inflammation-driven, successfully mimics the chronic, progressive process of the PD pathology in a natural, real exposure route for human. It is a unique addition to the current pool of animal models and provides important new insights into the inflammation-mediated chronic pathogenesis of PD and searching for therapeutic intervention in microglia-dopaminergic neuron pathway. 
